# Oxidative Stress at the Crossroads of Aging, Stroke and Depression

**DOI:** 10.14336/AD.2020.0225

**Published:** 2020-12-01

**Authors:** Anwen Shao, Danfeng Lin, Lingling Wang, Sheng Tu, Cameron Lenahan, Jianmin Zhang

**Affiliations:** ^1^Department of Neurosurgery, Second Affiliated Hospital, School of Medicine, Zhejiang University, Zhejiang, China.; ^2^Department of Surgical Oncology, Second Affiliated Hospital, School of Medicine, Zhejiang University, Zhejiang, China.; ^3^State Key Laboratory for Diagnosis and Treatment of Infectious Diseases, Collaborative Innovation Center for Diagnosis and Treatment of Infectious Diseases, The First Affiliated Hospital, College of Medicine, Zhejiang University, Zhejiang, China.; ^4^Burrell College of Osteopathic Medicine, Las Cruces, USA.; ^5^Center for Neuroscience Research, School of Medicine, Loma Linda University, Loma Linda, CA, USA.; ^6^Brain Research Institute, Zhejiang University, Zhejiang, China.; ^7^Collaborative Innovation Center for Brain Science, Zhejiang University, Zhejiang, China.

**Keywords:** oxidative stress, stroke, subarachnoid hemorrhage, intracerebral hemorrhage, depression, mitochondrial dysfunction, antioxidant, aging

## Abstract

Epidemiologic studies have shown that in the aging society, a person dies from stroke every 3 minutes and 42 seconds, and vast numbers of people experience depression around the globe. The high prevalence and disability rates of stroke and depression introduce enormous challenges to public health. Accumulating evidence reveals that stroke is tightly associated with depression, and both diseases are linked to oxidative stress (OS). This review summarizes the mechanisms of OS and OS-mediated pathological processes, such as inflammation, apoptosis, and the microbial-gut-brain axis in stroke and depression. Pathological changes can lead to neuronal cell death, neurological deficits, and brain injury through DNA damage and the oxidation of lipids and proteins, which exacerbate the development of these two disorders. Additionally, aging accelerates the progression of stroke and depression by overactive OS and reduced antioxidant defenses. This review also discusses the efficacy and safety of several antioxidants and antidepressants in stroke and depression. Herein, we propose a crosstalk between OS, aging, stroke, and depression, and provide potential therapeutic strategies for the treatment of stroke and depression.

## 1. Introduction

Epidemiologic studies demonstrate that 11% of the world’s population is over 60 years of age and this percentage will double to about 22% by 2050 [1]. A significant percentage of older individuals develop one or more age-related diseases, which may include two leading diseases characterized by high incidence and disability: stroke [2] and depression [3]. Stroke is classified into ischemic stroke and hemorrhagic stroke; the latter consists of intracerebral hemorrhage (ICH) and subarachnoid hemorrhage (SAH). It is estimated that on average, a person died from stroke every 3 minutes and 42 seconds in 2016 [4]. Global Burden of Disease 2017 identifies stroke as the third leading cause of years of life lost and disability-adjusted life years [5, 6]. Between 2006 and 2016, the actual number of stroke deaths increased 3.7%, although the age-adjusted mortality rate decreased 16.7% due to the large increase in the number of elderly people [4, 7]. Like stroke, another disease that affects a significant proportion of the population is depression, a psychiatric disorder characterized by inactivity and negative feelings of inadequacy. The 12-month prevalence of major depressive disorder (MDD) is about 6%, while the lifetime risk of MDD is nearly 15-18% [8-10]. Moreover, older age is identified as a consistent and important risk factor for a worse prognosis. This phenomenon may be associated with the effect of cognitive impairment [3]. As the World Health Organization (WHO) predicted depression to be the leading cause of global burden by 2030, attention should be paid to depression treatment [10, 11].

Over the past two decades, studies have identified the role of OS in these two diseases. Recently, preclinical experiments and clinical trials have focused on studying the efficacy of antioxidants and combined therapy with antidepressants in stroke or depressed patients. We review the results of experiments within the last 5 years, providing a comprehensive and novel overview of this interesting field.

**Figure 1.**
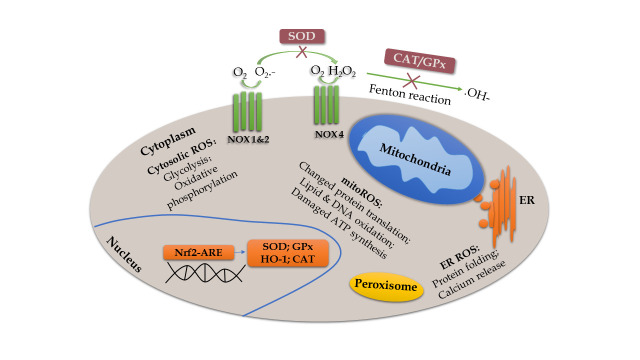
Schematic model of the main source of ROS and redox reaction. ROS are generated mainly from enzymatic reactions in the cytoplasm, endoplasmic reticulum, mitochondria, and peroxisome [300]. Specifically, overproduced mitoROS can affect metabolic pathways, such as alteration of protein translation, oxidation of lipid and DNA, and impairment of ATP synthesis [301]. Moreover, assembled NOX (NOX1 and NOX2) complex transports an electron from cytosolic NADPH to oxygen to form superoxide on the extracellular side [13]. The NOX4 complex rapidly converts the superoxide to H_2_O_2_, which undergoes a Fenton reaction to produce hydroxyl radicals and ions, and to regulate many downstream effects [302]. However, these oxidative events are inhibited by antioxidants, such as SOD and CAT/GPx. Activation of Nrf2-ARE pathway increases antioxidants, such as HO-1, SOD1, and CAT to protect cells from FR accumulation [303].

## 2. Overview of oxidative stress and the antioxidant system

OS describes a state in which the body produces excessive ROS and RNS in response to deleterious substances. Under normal conditions, OS and the anti-OS system are beneficial to physiological functions, such as respiration, circulation, etc. Mitochondrial dysfunction and internal environment disturbance can arise from multiple cellular molecules and signaling pathways as a result of imbalanced redox reactions, characterized by increased production of reactive oxygen species (ROS) and reactive nitrogen species (RNS) (Fig. 1), as well as decreased antioxidant defenses. The most important ROS are mitochondrial ROS (mitoROS) produced from enzymes of the mitochondrial respiratory chain. Overproduced mitoROS can affect metabolic pathways, leading to a compromised function of organelles [12]. Another significant source of ROS is NADPH oxidase (NOX). When the complex of NOX is assembled, it transports an electron from the cytosolic NADPH to oxygen to then form superoxide on the extracellular side [13]. The antioxidant system consists of enzymes and nonenzymatic substances, among which nuclear factor erythroid 2-related factor (Nrf2) is the most important factor. Nrf2 is a transcription factor and can recognize the antioxidant response element (ARE) to regulate several genes, such as heme oxygenase 1 (HO-1). Activation of the Nrf2-ARE pathway increases antioxidants and protects the cells from free radical (FR) accumulation [14].

Under physiological conditions, moderate OS activity is necessary for body health. Toxic effects derived from ROS and RNS can be ameliorated or neutralized by FR scavengers and the antioxidant system. However, when a large number of ROS and RNS are generated, excessive FR then induce molecular oxidation, cell membrane modification, and enzyme inactivation, resulting in cellular damage and functional decline [15]. Overactive OS with an imbalanced redox state can induce many injuries, especially to the brain, partly expounding the role of OS in both stroke and depression.

## 3. Overview of stroke and depression

**Figure 2.**
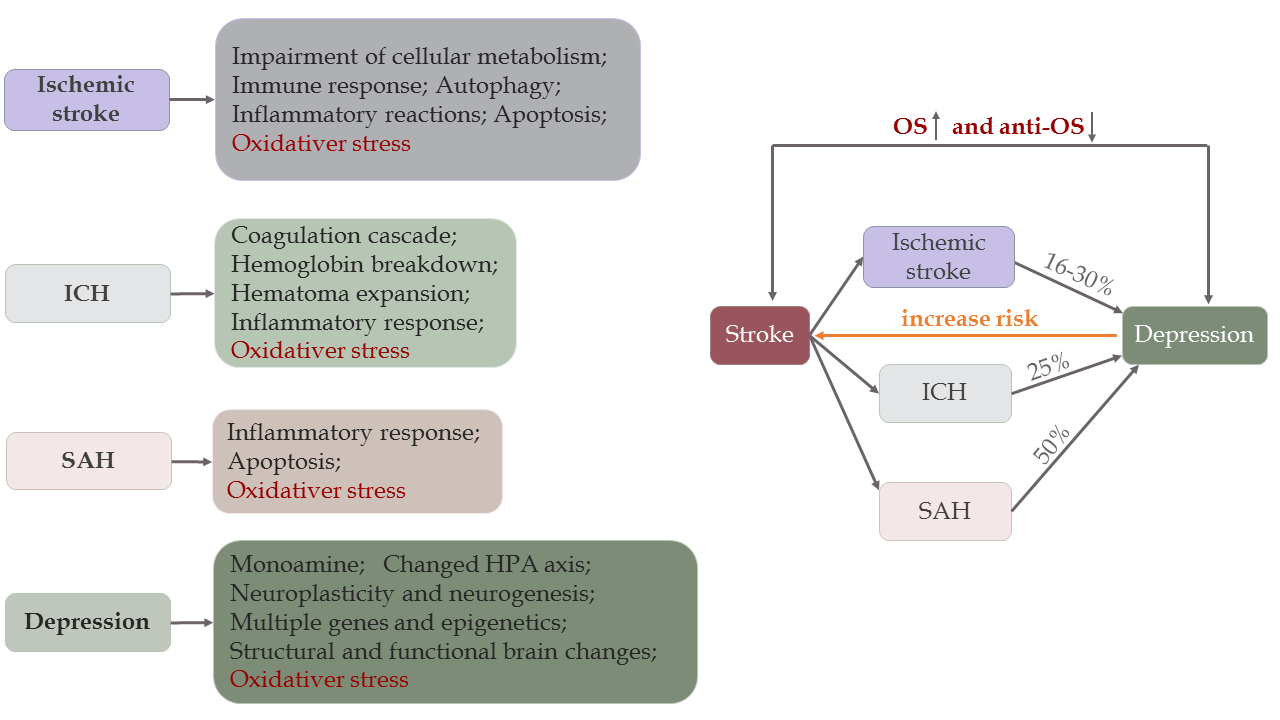
Pathogenesis and correlation between stroke and depression. There are different mechanisms in ischemic stroke [16], ICH [17], SAH [304], and depression [10]. Stroke and depression are associated with oxidative stress. Due to overactive OS activity and impaired anti-OS defenses, 16-30% of ischemic stroke survivors [31, 32], 25% of ICH [33] patients, and 50% of SAH [34] patients may develop depression later, but the age groups vary among studies. Conversely, depression increases the risk of stroke by 33% in patients who experience stressful life events [37].

Ischemia-hypoxia often occurs in ischemic stroke, while intracerebral bleeding in the brain parenchyma can be found in ICH, and blood clots can be found in the subarachnoid space of SAH patients. Once cerebral ischemia occurs, it impairs cellular metabolism and triggers pathological pathways, such as immune responses, inflammatory reactions, OS, autophagy, and apoptosis, leading to irreversible neuronal damage and brain injuries accompanied by blood-brain barrier (BBB) disruption (Fig. 2) [16]. Particularly, the release of FR further damages the brain. Different from ischemia, both primary and secondary brain injury (SBI) occur in ICH and SAH. Mechanisms participating in post-ICH include the coagulation cascade (particularly thrombin), hemoglobin degradation, inflammation, apoptosis, necrosis, OS, and hematoma expansion [17]. Post-ICH events lead to SBI, which manifests as brain edema, BBB disruption, brain atrophy, vasospasm, neurological deficits, and even death [17]. Compared with ischemic stroke, the results of OS activity in the studies were similar in ICH. Indeed, this kind of behavior exhibited by OS also occurs in SAH [18]. Furthermore, brain injury involved in SAH is divided into two stages: an early stage within the first 72 hours, called early brain injury (EBI), and the delayed stage. OS plays a direct and indirect role in both stages, and many recent studies demonstrate efficacy of antioxidants in animal models, especially in EBI [19, 20].

In comparison with stroke, the main factors promoting MDD are psychosocial stressors, and the main pathophysiology of depression is associated with decreased monoamine levels, an altered hypothalamic-pituitary-adrenal axis [21], inflammation [22], neuro-plasticity and neurogenesis controlled by BDNF [22], and structural and functional brain changes (Fig. 2) [23, 24]. Apart from inflammation and neurogenesis, OS also plays a crucial role in depression. The role of OS in depression is supported by many studies that show upregulated OS activities and downregulated anti-OS responses [25-27]. In addition, the antioxidants may ease depressive symptoms [28, 29]. In summary, there is a close relationship between depression and OS.

As mentioned above, OS not only exists in stroke, but also occurs in depression. Moreover, many clinical trials have proved that stroke increases the incidence of depression, which inversely acts as an independent risk factor for stroke. On one hand, both ischemic and hemorrhagic stroke increases the prevalence of depression. A multicenter study found that approximately one-fifth of patients developed depression after stroke at a 12-month follow-up [30]. In addition, 757 patients with first-ever minor ischemic stroke were followed for one year and analysis showed that nearly 30% of patients developed depression [31]. However, the incidence rates of depression in ischemic stroke survivors aged 18-50 years and in the control group were 16.8% and 6.1%, respectively [32]. A similar relationship is also seen in ICH [33] and SAH [34]. On the other hand, an Australian longitudinal study with a 12-year follow-up suggested that depression caused a 2-fold increase in the odds of stroke [35]. In addition, Pan did a meta-analysis consisting of 28 prospective cohort studies illustrating that the risk of ischemic stroke was exacerbated by depression [36], whereas another meta-analysis found that patients experiencing stressful life events had a 33% increased risk of total stroke [37] (Fig. 2).

## 4. Roles of oxidative stress in stroke and depression

Following ischemic stroke, an excess of ROS and RNS causes an ascending concentration of H^+^ and H_2_O_2_, leading to DNA damage, endothelial impairment, and mitochondrial dysfunction (see below for details). Furthermore, they exacerbate the damage through OS-mediated inflammation, apoptosis, autophagy, and the microbial-gut-brain axis. Apart from the mechanisms shown in ischemic stroke, the ROS and RNS, derived from the hemoglobin-heme-iron axis and activation of NOX, increase glutamate and inflammatory activities in ICH, and they disrupt BBB integrity to influence EBI and SBI in SAH. It is unsurprising to find semblable outcomes and pathological processes of OS in depression.

### 4.1 Oxidative stress in stroke and depression

**Figure 3.**
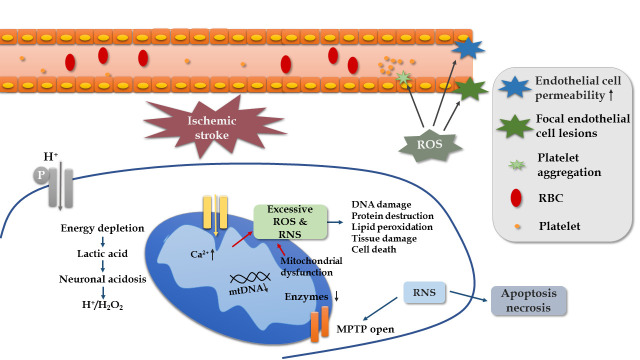
Mechanism of oxidative stress in ischemic stroke.

Because the human brain requires constant oxygen and nutrients to maintain its function, it is vulnerable to FR attack. The depletion of energy in ischemic stroke can cause a series of damage to promote the development or recurrence of stroke, and OS is involved in all stages of ischemic stroke progression (Fig. 3). Firstly, energy expenditure leads to the accumulation of H^+^ concentration and H_2_O_2_. Studies on a mouse model of focal ischemia found that extracellular proton induce neuronal necroptosis via acid-sensing ion channel 1a/receptor interaction protein 1 association [38]. Secondly, ROS have effects on cerebral blood flow. ROS stimulates vasoconstriction and increases platelet aggregation and endothelial cell permeability, thereby affecting blood circulation [39]. Thirdly, RNS play a role in mitochondrial functions, such as reducing DNA and suppressing enzymes of the mitochondria. This is favored by a case-control study, in which a low mitochondrial DNA content in peripheral blood leukocytes is significantly related to ischemic stroke [40]. Lastly, ROS and RNS bring about DNA damage, protein destruction, lipid peroxidation, and cell death, leading to poor outcomes [41, 42]. The results of clinical studies have demonstrated that higher plasma levels of oxidized low-density lipoprotein reveal a worse prognosis [43], a higher prevalence of cognitive impairment [44], and an increased risk of death [45].

**Figure 4.**
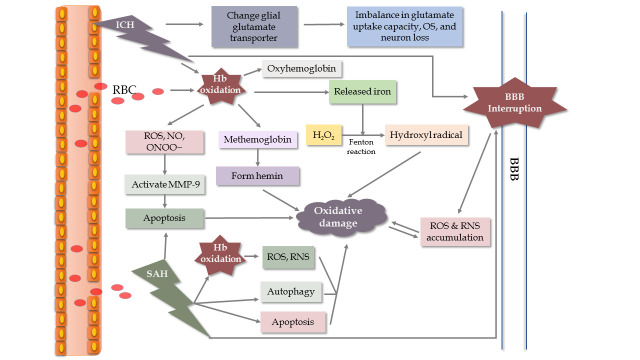
Schematic model of oxidative mechanisms in ICH and SAH, especially associated with hemoglobin (Hb). During the hemoglobin-heme-iron axis, Hb is released into the extracellular space and is accompanied by an abundance of superoxide generated from the non-enzymatic oxidation of Hb [46]. This oxidation of Hb produces methemoglobin, which releases heme to stimulate lipid peroxidation and other oxidative actions around the hematoma in brain tissue. Meanwhile, iron released from Hb degradation is used in the Fenton reaction to transform H_2_O_2_ into the hydroxyl radical, leading to increased oxidative damage [47].

Hemorrhagic stroke is a common, serious neurological disease associated with high disability and mortality, especially when associated with ICH. One of the main underlying mechanisms is OS. ROS and RNS usually come from the metabolite axis of hemoglobin-heme-iron, NOX activation, increased glutamate and inflammatory activities. Upon the onset of ICH, bleeding into the brain parenchyma is commonly observed. Then, several biological events associated with hemoglobin oxidation and released iron occur, resulting in neurological damage (Fig. 4) [46, 47]. What’s more, the results suggest that iron chelators could attenuate ROS generation to improve neurological function following ICH, further supporting the hemoglobin-heme-iron axis [48]. Additionally, there are other mechanisms participating in the process of ICH, such as glial glutamate transporter responses [49] and mitochondria-dependent apoptosis [50]. The effects caused by other pathological events are similar to those in ischemic stroke [51, 52]. Besides neuronal injuries, those pathological events contribute to SBI, including brain edema, BBB breakdown, and vasospasm. Interruption of the BBB facilitates ROS and RNS accumulation, which amplifies brain injuries [53].

In SAH, EBI is identified as the immediate injury accompanied by subsequent events (brain edema, inflammation, apoptosis, etc.) in the first 72 hours, and EBI plays a vital role in the pathological processes (Fig. 4). After the occurrence of SAH, the intracranial pressure will rise to the pressure of the diastolic artery, which reduces cerebrospinal fluid pressure and disturbs cerebral autoregulation before BBB breakdown and occurrence of cerebral edema [54]. Due to erythrocytosis, the central nervous system (CNS) is exposed to high levels of Hb and Hb-degradation products in the subarachnoid space. This pathological process produces excess ROS and RNS, and promotes cerebral vasospasm, cerebrovascular stenosis, and delayed cerebral ischemia [55]. Furthermore, several harmful events occur in SAH survivors, including altered ionic homeostasis, excitotoxicity, destruction of vascular integrity, OS, inflammation, apoptosis, autophagy, and activation of the NOS pathway [56-62].

The impact on stroke pertains to an abundance of ROS and RNS, as well as OS-induced cell death pathways. So, what role could OS have in depression? Depression involves several pathological processes that include an imbalance of neurotransmitters, inflammation, OS, apoptosis, glutamate excitotoxicity, and the microbiota-gut-brain axis [10, 63-65]. Although there is a deficiency of direct evidence exhibiting mechanisms between OS and depression, numerous animal and human studies have reported functions of OS in depressed patients via various OS-associated molecules [66-69]. For example, Pasquali et al. conducted a longitudinal study and described a series cascade of pro-oxidative and pro-inflammatory events contributing to the development of MDD in middle-aged women [70]. Besides hyperactive OS, reduced antioxidant activity may exacerbate depression, but antioxidant treatment shows anti-depressant effects [71-74]. Wigner et al. proved that the polymorphisms of antioxidant enzymes (e.g., superoxide dismutase (SOD) and catalase (CAT) or glutathione peroxidase (GPx)) could regulate the risk of depression [72]. Parallel results in the clinical trial were found to reach a consistency in the effects of OS in depression [75].

### 4.2 Common oxidative stress-mediated process between stroke and depression

**Figure 5.**
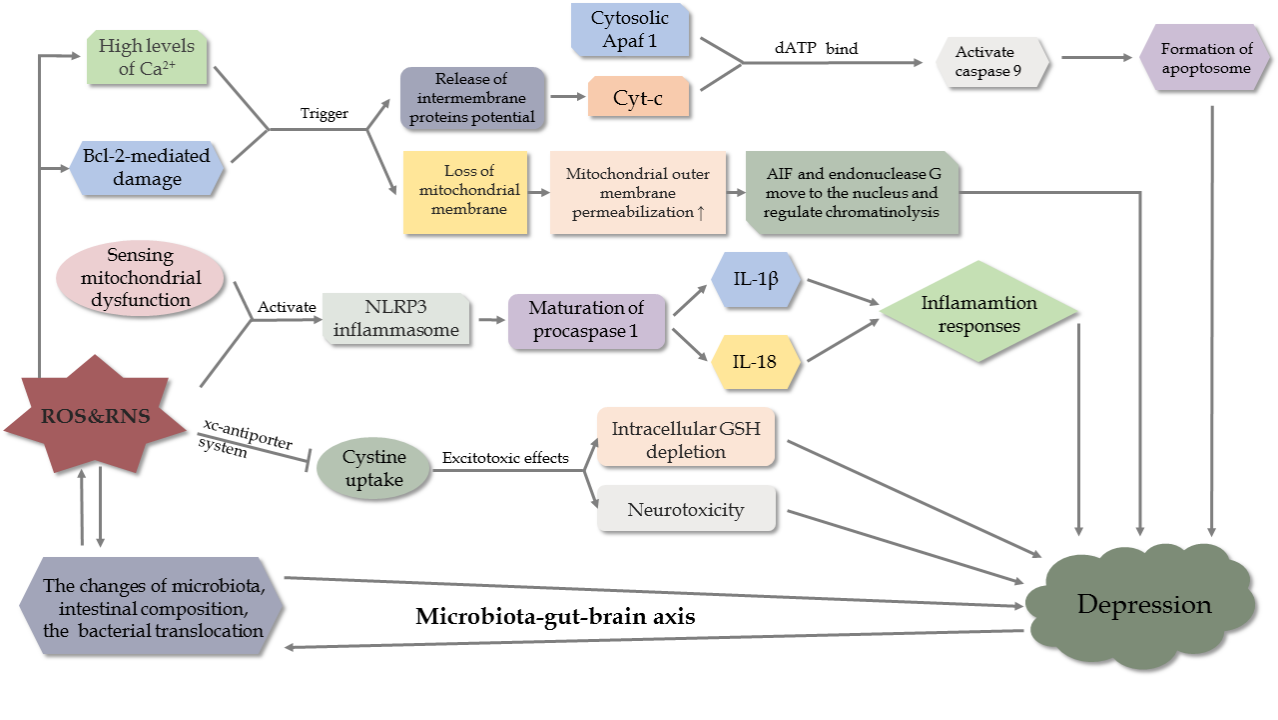
Mechanisms of oxidative stress and OS-mediated cell death pathway in depression.

In previous sections, it is mentioned that OS-mediated pathological mechanisms (e.g., inflammation and apoptosis) act as a bridge and exert functions in stroke and depression (Fig. 5). Details about common OS-induced mechanisms are shown as follows.

#### 4.2.1 Oxidative stress-mediated inflammation

Overproduction of ROS and RNS can activate inflammatory processes to aggravate brain damage through glycogen synthase kinase 3 (GSK-3) and endothelial injury. ROS stimulate the phosphorylation of GSK-3 to negatively affect the cyclic adenosine monophosphate response element-binding protein (CREB) by suppressing its nuclear translocation, resulting in an increased expression of pro-inflammatory cytokines and brain dysfunction in ischemic rat [76, 77]. To be specific, activated GSK-3 downregulates Nrf2-ARE binding activity and decreases the expression levels of genes downstream of Nrf2-ARE [78], but it stimulates the Toll-like receptors (TLRs) in the peripheral blood mononuclear cells [79]. Experiments indicate that TLR 4 induces the expression of inflammatory elements through activation and nuclear translocation of NF-κB, while deletion of TLR 4 attenuates ischemic cerebral injury [80]. Additionally, GSK-3 could boost inflammation by interrupting CREB binding proteins, which are the co-activators of CREB and NF-κB in the nucleus [76, 81]. Inversely, inhibition of GSK-3β improves the transcription and expression of anti-inflammatory cytokines and reduces pro-inflammatory cytokine production [79]. Therefore, the results of GSK-3 activation and GSK-3 suppression confirm the role of OS-induced inflammation in ischemic brain injury. In addition to GSK-3, oxidative injury of platelet and endothelium cells also participate in inflammation. Following endothelial impairment, P-selectin is highly expressed to induce the rolling of leukocytes on the endothelium, facilitating stable adhesion between leukocytes and endothelial cells [82, 83]. Afterwards, the adhered leukocytes release matrix metalloproteinase, break down the BBB, and enter into the brain parenchyma to trigger a series of inflammatory processes [84]. In ICH, a similar process occurs [51]. In SAH, free Hb stimulates endothelial cells to express cell adhesion molecules to attract neutrophils. Cells trapped in the subarachnoid space then undergo an oxidative burst, which releases ROS-mediated inflammatory cytokines or other molecules to further damage the brain [56].

In depressed individuals, ROS and RNS have effects on inflammatory reactions via NOD-, LRR- and pyrin domain-containing protein 3 (NLRP3)[85]. After sensing mitochondrial dysfunction of ROS, activated NLRP3 inflammasome induce the maturation of procaspase-1, which is an initiating process of interleukin (IL)1β and IL-18, to initiate inflammatory responses. Nevertheless, IL-1β knock-down in the hippocampus attenuate depression-like behaviors induced by LPS in mice [86]. However, these changes are reversed by treatment with amitriptyline [87, 88]. In addition, pattern recognition receptors recognize PAMPs and DAMPs to trigger MAPK and/or NF-κB to activate immune-inflammation [89, 90].

#### 4.2.2 Oxidative stress-mediated apoptosis

Accumulating evidence has shown that apoptosis, necrosis, and their combined pathway, “necroptosis”, participated in ischemic stroke stimulated by ROS and RNS [91]. Experimental data obtained from ischemic mouse models suggest that the nuclei of neurons exhibit apoptotic morphology after four hours of ischemia [92]. Mechanistically, under pathological conditions, cellular stress stimulates an apoptotic signaling pathway to activate the caspase protease and cause mitochondrial dysfunction, resulting in characteristic changes of apoptotic cell morphology, such as cell rounding, plasma membrane blebbing, and nuclear fragmentation. Apoptosis can be induced by extrinsic and intrinsic stimuli. The extrinsic stimuli are triggered by the death receptor signaling pathway [93], while the intrinsic stimuli involve a mitochondrial signaling pathway and released cytochrome c (Cytc) [94]. When ischemic stroke occurs, OS initiates the apoptosis pathway mainly through intrinsic stimulation. OS also regulates apoptotic activities through modulating the balance between the anti-apoptotic protein, Bcl-2, and the pro-apoptotic regulator, Bax protein [94]. In ICH, Cytc-mediated and mitochondria-dependent apoptosis is also demonstrated to be an important part of the OS-mediated mechanisms. Additionally, Hb-induced ROS, NO, and peroxynitrite (ONOO-) are shown to directly or indirectly activate matrix metalloprotein 9, leading to apoptosis [95, 96]. Meanwhile, there is a significant increase in the number of apoptotic neural cells in the rat brain after SAH [60].

Parallel to OS-mediated apoptosis in stroke, intrinsic apoptosis associated with mitochondria occupies an important position in the development of depression. When exposed to chronic stress, overactive apoptosis stimulated by OS in neurons promotes the neurodegeneration in depressed animals [97]. Mechanistically, Bcl-2-mediated damage and excess levels of Ca^2+^ trigger various kinases, leading to a loss of the mitochondrial membrane potential, and an increased release of intermembrane proteins into the cytoplasm. In the presence of dATP, the released Cytc binds to the cytosolic Apaf 1 to activate caspase-9 and promote apoptosome formation. Meanwhile, enhanced permeability of the outer membrane of the mitochondria allows apoptosis-inducing factor (AIF) and endonuclease G to move to the nucleus and regulate chromatinolysis [97]. In addition, chronic mild stress suppresses the expression of Bcl-2-associated athanogene-1, a gene involved in anti-apoptosis, resulting in the activation of caspases, BAX, and Bcl-2 antagonists in the mitochondria. Consequently, there is an excess of neuronal death [98]. Moreover, it is highlighted that polymorphisms of apoptotic protein genes may be associated with MDD [99].

#### 4.2.3 Oxidative stress-mediated microbiota-gut-brain axis

Recently, the microbiota-gut-brain axis has gained extensive attention as a channel for communication and physiological regulation. Activities of the intestinal microbiome might promote abnormal protein aggregation and oxidative responses to impair the brain. Inversely, the brain can either directly or indirectly impact commensal organisms or gastrointestinal functions through the release of signaling molecules from cells in the lamina propria [100, 101]. After an ischemic stroke, there is an increase of gastrointestinal complications such as dysphagia [102], fecal incontinence [103], gastro-intestinal hemorrhage ^[104]^, and constipation [105]. Wen et al. revealed that ischemic stroke exacerbates gut barrier breakdown and microbiota alterations. Subsequently, the translocation of a selective bacterial strain from the host intestinal microbiota to the surrounding tissues promotes post-stroke infections [106]. Furthermore, Benakis C et al. suggested that intestinal dysbiosis disequilibrates the intestinal immune homeostasis by inhibiting interleukin-17-positive γδ T cells, and suppressing the trafficking of effector T cells from the gut to the leptomeninges [107]. Moreover, vagal afferent innervation of the enterochromaffin cells provides direct signaling to the neuronal circuits, leading to changes of the immune-response and other homeostatic functions [108]. Due to lack of data showing the relationship the among gut-brain axis and ICH or SAH, further studies are needed.

In depression, the changes of microbiota, intestinal composition, and the resultant bacterial translocation expand the content of two-way interaction of the gut-brain axis. Zhang P et al. recognized the gut microbiome as an environmental factor that can shape the brain through this axis. Data from clinical sampling showed a significant difference of gut microbiota compositions between MDD patients and healthy controls, demonstrating that gut dysbiosis may mediate the host's metabolism to promote depressive-like behaviors [109]. In addition, gut dysbiosis could have an impact on the biology of MDD through several pathways involving immune activation, ROS and RNS, and neuroplasticity cascades [110]. Therefore, the microbiota-gut-brain axis may play a key role in the pathophysiological process of MDD. This axis opens a new preventative and therapeutic perspective for depression. However, due to methodological inconsistencies and limitations, more fundamental and clinical research should be undertaken.

## 5. Roles of aging in stroke and depression via oxidatvie stress

The preceding content shows us a close connection between stroke and depression via the OS system and OS-mediated biological processes. Moreover, numerous studies have proved that aging or senescence is a risk factor that aggravates stroke and depression. For example, the population-based multiethnic cohort reported that the risk effect of physical inactivity is modified by age, and there is a conspicuous risk found only in stroke patients >80 years of age [111, 112]. Population-based surveillance studies predicted that the number of strokes will double by 2050, with incidence occurring primarily in the elderly (>age 75) groups [113]. Although depression prevalence in the elderly varies across studies, Sjoberg L et al. indicated that older adults with poor physical function have a higher prevalence of depression [114]. Herein, there is an “aging-stroke-depression” network and OS seems to be the center of this network. So, what relationship does aging have with oxidative stress, stroke, and depression?

### 5.1 Aging and oxidative stress

There is a close link between oxidative stress and aging, and this link can be proven through many related mechanisms [115, 116]. Firstly, age-related cognitive decline is a consequence of increased OS and neuroinflammation activity in the the aging hippocampus, and a consequence of reduced neurogenesis and synaptic plasticity [117]. Furthermore, mutual effects of inflammation and OS are observed to exacerbate the aging brain. Inflammation stimulates both macrophages and microglia to generate mitoROS to cause cognitive decline, whereas OS-damaged cells produce inflammatory mediators to promote microglial aging [118]. Secondly, aging and OS can damage the brain by negatively affecting neuroplasticity, brain homeostasis, and cognitive function [119, 120]. The third factor is glutathione. In animal experiments, glutathione deficiency might compromise the ability of the aging brain to meet the demands of OS, leading to impaired physiological functions [121].

### 5.2 Aging and stroke

Normally, physiological events of aging involve a decline of innate functions, including shortened telomeres, dysregulated hormones, and dampened immune responses. Under pathological conditions, aging stimulates and exacerbates cellular injuries, such as DNA damage and FR accumulation, to increase the vulnerability of the brain [122]. Mattson MP et al. depicted ten hallmarks of brain aging (e.g., OS and impaired DNA repair), among which, dysregulated energy metabolism is the core factor [122]. When exposed to ischemia, aging impairs the integrity of the neurovascular unit and damages brain tissues [123]. Aging can also destroy collateral circulation and revascularization of the brain through increased FR and inflammatory responses to aggravate stroke [124]. Age-related cognitive deteriorations also exist in the ICH mice model. Researchers showed a delayed neurological improvement and decreased levels of antioxidants in senescence-accelerated mice than senescence-accelerated resistant mice, though the time period in which the neurological deficits occurred and increased remains the same. To conclude, these results implied a key role of OS in senescence and stroke.

Apart from OS, several other mechanisms are involved in OS and stroke. The ubiquitin proteasome pathway protects neurons by removing abnormal or toxic proteins located in the axons and dendrites, and ischemia may exacerbate injury of an aged brain through impairing ubiquitin proteasome function [125]. Furthermore, aging is detrimental to ischemic stroke recovery due to its role in altering astrocytic proliferation, inhibiting vascular endothelial growth factor (VEGF) production, and upregulating the release of inflammatory cytokines [126, 127]. What about aging in hemorrhagic stroke? Apart from the direct effects of aging in ICH, senescence may contribute to ICH progression by changing the body physiology and increasing the risk of multiple chronic health conditions and comorbidities, such as hypertension and diabetes [128]. However, the exact mechanism remains unclear. To our knowledge, there is no clear evidence explaining a relation between SAH and aging.

### 5.3 Aging and depression

In the process of aging, biological changes in depression include mitochondrial dysfunction, dopamine dysfunction, and increased proinflammatory cytokines involved in the cellular senescence cycle. In general, mitochondrial abnormalities can be measured in peripheral blood mononuclear cells [129]. For instance, the clinical study by Karabatsiakis et al. depicted higher levels of mitochondrial impairments in older folks with depression when compared with healthy controls [130]. A systematic review and meta-analysis also supported this connection, indicating that late-life depression may be associated with a decreased hippocampal volume, though the relationship was not straightforward [131].

Different from other mechanisms in stroke, the pathological process of depression during aging is significantly affected by dopamine dysfunction, inflammation, and psychological stressors. In regards to dopamine, the availability of D2 receptors in the caudate and shell nuclei is attenuated in older adults, leading to decreased motor speed and impaired frontal functioning [132]. Worse still, dopamine levels in the striatum of the elderly are only 40% of those found in young adults, and the D1/D2 receptor density and dopamine transporter expression decreased approximately 10% per decade, throughout the life cycle [133, 134]. Secondly, studies have found that inflammatory processes, especially high expression levels of IL-6, TNF-α, and C-reactive protein contribute to the depressive symptoms in elderly adults, with clinical manifestations of lethargy, slowing, and weakness [135]. Most importantly, psychological well-being is largely associated with the onset and progression of depression among the elderly population [136]. As the relationship between physical health and subjective well-being is bidirectional, elderly people with chronic diseases (e.g, coronary heart disease and chronic lung disease) have higher levels of unhappiness and are more likely to have depression [136]. Fourthly, a cross-sectional survey showed that malnutrition increased the prevalence of depression in the elderly Nepali population. [137]. Lastly, low folate levels result in increased mild cognitive impairment, dementia, and depression in older individuals [138]. In summary, aging and depression are closely linked through OS, OS-related inflammation, and dopamine dysfunction, although social life and malnutrition play a crucial role in this link.

### 5.4 Aging and oxidative stress in stroke or depression

**Figure 6.**
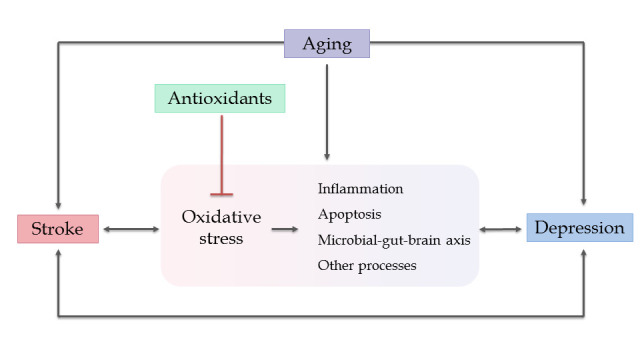
Proposed crosslink and interplay among aging, oxidative stress, stroke and depression.

There are some changes of molecules and signaling pathways in aging. Under ischemia/hypoxia conditions among elderly individuals, silent information regulator 1 (SIRT1) expression and the mitochondrial unfolded protein response are reduced with age, leading to impairment of mitochondrial function [139]. This condition also occurs in another study, indicating that the post-translational regulation of molecular mediators, such as hypoxia-inducible factor 1α and SIRT1, and the glycolytic-mitochondrial energy axis are critical in response to hypoxic-ischemic injury [123]. Moreover, E2f transcription factor 1 enhances the cellular senescence in human fibroblast cells, while transcription factor FOXO3 plays against senescence by regulating ROS scavenging proteins. E2f transcription factor 1 could inhibit FOXO3-dependent transcription by directly binding to FOXO3 in the nucleus to expedite the aging process [140]. Interestingly, the telomerase reverse transcriptase (TERT), a catalytic subunit of telomerase, exerts neuroprotective effects in the mitochondria of neurons by decreasing the ROS and protecting DNA. Nevertheless, recent studies observe the neuroprotective effects of TERT in cellular and animal models after aged brain injury, but it is unclear about TERT’s effects in the human CNS [141]. In regards to aging, OS, and depression, glutathione captures our attention. In a study enrolling 58 depressed older patients and 12 controls, glutathione was measured in the anterior cingulate cortex, and the data showed that increased glutathione/creatine ratios are associated with greater depressive symptoms than the control group [142]. In conclusion, OS lies in the center of the “aging-stroke-depression” network (Fig. 6). First, when stroke occurs in animals or patients, excessive generation of ROS follows, leading to cellular damage and brain injury. Second, OS mediates inflammation, apoptosis, and the microbiota-gut-brain axis to increase the accumulation of ROS, followed by brain deterioration. Third, aging acts as a risk factor and aggravates the development of stroke and depression via OS and OS-induced pathways. Due to the central role of OS in this network, administration of antioxidants seems to provide therapeutic ways for stroke and depression. So, are antioxidants safe and effective in these two disorders?

## 6. Antioxidants and antidepressants in stroke/depression

In the last 5 years, mountains of preclinical and clinical studies have found that the application of antioxidants inhibits stroke and depression in patients with either, or both diseases, although the therapeutic effects and safety are under consideration. What’s more, the main pharmacotherapy for depression patients focuses on antidepressants [143], and the selective serotonin reuptake inhibitors (SSRIs) have been used as first-line antidepressants in recent decades. However, SSRIs have shown moderate efficacy and significant side-effects [10]. Variability in efficacy and acceptability are large in head-to-head trials [144]. Therefore, development of an accurate and effective antidepressant regimen requires our constant efforts. Herein, we list most of the relative studies about antioxidants and antidepressants used in treating stroke/depression, in order to find potential therapies.

### 6.1 Antioxidants in stroke

Antioxidants or anti-oxidative systems have neuroprotective effects through different anti-activities and various signaling pathways, especially the Nrf2 pathway found in either ischemic stroke or hemorrhagic stroke (Table 1). Firstly, antioxidants alleviate ischemic stroke by attenuating the OS activity, and by protecting the mitochondrial function and BBB. For example, fucoxanthin [145], Sirtuin 6 [146], protocatechualdehyde [147], mangiferin [148], Korean Red Ginseng [149], 11-Keto-β-boswellic acid [150], metformin [151], s-allyl cysteine [152], monomethyl fumarate[153], dieckol [154], and fumarate [155] are reported to protect the brain by activating the Nrf2/HO-1 pathway. As such, uric acid (UA) [156], HP-1c [157], andrographolide [158], 2,2,6,6-tetramethyl-1-piperidinoxyl [159], and 3H-1,2-Dithiole-3-thione are associated with Nrf2 [160]. Secondly, protecting mitochondrial function is beneficial to ischemic stroke treatment, such as 3-n-butylphthalide [161] and 5-methoxyindole-2-carboxylic acid [162].

Melatonin activates the silent information regulator 1 signaling [163], and progesterone suppresses mtROS production and blocks MPTP, to exert mitochondrial protection [164]. SkQR1 is a mitochondrial-targeted antioxidant, and it is effective in protecting newborn brains before and after ischemic stroke [165]. Thirdly, antioxidant systems also exert protective effects on blood vessels. For instance, ginkgolide [166] and leonurine [167] promote angiogenesis via upregulated VEGF expression. Fourthly, BBB integrity can be protected by several antioxidants, including astragaloside IV [168], Osthole [169] and the Chinese herbal medicine formula, Tao Hong Si Wu Decoction[170, 171]. All of these antioxidants are correlated with Nrf2 signaling.

**Table 1:** Antioxidants in Stroke.

Stroke	Antioxidants	Anti-OS activity	Mechanism of anti-OS activity and others
Ischemic stroke	fucoxanthin	anti-OS	inhibit OS via Nrf2/HO-1 signaling pathway
	Sirtuin 6		
	protocatechualdehyde		
	mangiferin		
	Korean Red Ginseng		
	11-Keto-β-boswellic acid		
	metformin		
	S-allyl cysteine		
	monomethyl fumarate		
	dieckol		
	fumarate		
	ursolic acid		upregulate Nrf2 pathway and expression levels of BDNF
	HP-1c		AMPK-Nrf2 pathway activation, without any toxicity after penetrating the brain
	andrographolide		up-regulate Nrf2/HO-1 expression via regulation of p38 MAPK
	2,2,6,6-tetramethyl-1-piperidinoxyl		inhibit p38 MAPK and p53 cascades
	3H-1,2-Dithiole-3-thione		suppress microglia activation; inhibit CNS peripheral cell infiltration
	3-n-butylphthalide	anti-OS; attenuate mitochondrial dysfunction	inhibit OS; activate Nrf2/HO-1/AMPK pathway; improve MMP and complexes I-IV
	melatonin		activate SIRT1 signaling
	progesterone		suppress mtROS production and block MPTP
	5-methoxyindole-2-carboxylic acid		increase antioxidative capacity via the Nrf2 signaling pathway; reduce OS
	SkQR1		protect mitochondria
	GK	anti-OS; protect blood vessels	faciliate angiogenesis through HIF-1α/VEGF and JAK2/STAT3 pathway
	leonurine		upregulate VEGF expression by Nrf-2 pathway
	astragaloside IV	anti-OS; protect BBB	Nrf2 signaling pathway
	Tao Hong Si Wu Decoction		anti-OS
	schizandrin A	anti-OS; anti-inflammation	AMPK/Nrf2 pathway
	tryptanthrin		decrease pro-inflammatory cytokines in BV2 microglia cells via Nrf2/HO-1 signaling and NF-κB
	3, 14, 19-triacetyl andrographolide		inhibit TLR4/NF-κB; upregulate Nrf2/ARE
	quercetin		suppress LPS-induced oxidant production and expression of adhesion molecules
	apelin 13		affect AMPK/GSK-3β pathway activated by AR/Gα/PLC/IP3/CaMKK signaling;
	diosgenin		suppress TLR4/MyD88/NF-kB-induced inflammation
	irisin		regulate ROS-NLRP3 inflammation
	TPEN		inhibit OS and inflammation
	N-acetyl lysyltyrosylcysteine amide		
	Tanshinone IIA		
	berberine		reduce the infarct volume and brain edema; improve motor function;
	melilotus officinalis		reduce cerebral thrombosis and inflammatory mediators
	DHC		protect BBB; inhibit inflammation by affecting ROS, NOX2, NOX4, NF-ĸB, and NO
	resveratrol		modulate intestinal flora-mediated Th17/Tregs and Th1/Th2 polarity shift
	EPO-cyclosporine		suppress the innate immune response to OS, inflammation and MAPK family signaling
	rhein	anti-OS; anti-apoptosis	inhibit OS and apoptosis
	deuterohemin His peptide-6		
	acteoside		
	radix scrophulariae		
	pomalidomide		
	clostridium butyricum		
	adiponectin		attenuate mitochondrial vulnerability through the JAK2/STAT3 pathway
	YiQiFuMai		reduce PKCδ/Drp1-mediated mitochondrial fission
	withania somnifera		inhibit PARP1-AIF-Mediated caspase-independent apoptosis
	SMXZF		suppress H2O2-induced neuronal apoptosis through caspase-3/ROCK1/MLC pathway
	diallyl trisufide		suppress OGD-induced apoptosis via the PI3K/Akt-mediated Nrf2/HO-1 signaling pathway
	plumbagin	anti-OS; anti-inflammation; anti-apoptosis	inhibit OS, inflammation and apoptosis
	hollow prussian blue nanozymes		
	geniposide		
	curcumin		
	hexahydrocurcumin		
	Sirt3	promote autophagy	regulate the AMPK-mTOR pathway; decrease H2O2; increase ATP generation
	β-arrestin-1		interact two major components of the BECN1 autophagic core complex
	vitexin	anti-autophagy	inhibit autophagy through the mTOR/Ulk1 pathway
	silibinin		suppress the mitochondrial and autophagic cell death pathways
	3-methyladenine		inhibit expression of LC3 and Beclin-1
	astragalosides		block OGD-R-induced apoptosis and autophagy by inhibiting OS and ER stress
	isoquercetin	anti-OS; anti-inflammation; anti-apoptosis; anti-autophagy	influence TLR4, NF-κB and caspase-1; ERK1/2, JNK1/2, and MAPK; TNF-α, IL-1β and IL-6; NOX4/ROS/NF-κB signaling pathway; CREB, Bax, Bcl-2, and caspase-3
	ECGG		affect PI3K/AKT/eNOS and NRF2/HO-1 signaling pathway; promote neovascularization and cell proliferation
ICH	green tea	anti-OS	reduce EBI
	cofilin		
	mammalian sterile 20-like kinase-1		
	melatonin		reduce DNA damage and MPTP opening
	dexmedetomidine		inhibit PGC-1α pathway inactivation and mtROS
	oleuropein		alleviate brain edema; preserve the BBB
	adiponectin		
	isoliquiritigenin	anti-OS; anti-inflammation	ROS/NF-κB, NLRP3 inflammasome pathway and Nrf2-mediated activities
	Sirt3		suppress NLRP3 and IL-1β levels
	Sodium Benzoate	anti-OS; anti-apoptosis	regulate DJ-1/Akt/IKK/NFκB pathway to inhibit neuronal apoptosis and mtROS
	carnosine		decrease brain edema, BBB disruption, OS and neuronal apoptosis
	metformin	anti-OS; anti-inflammation; anti-apoptosis;	inhibit OS, apoptosis and neuroinflammation
	baicalein		
	hydrogen gas		
	protocatechuic acid		
	hypoxia-inducible factor prolyl hydroxylase domain (HIF-PHD) metalloenzymes		abolish ATF4-dependent neuronal death
SAH	dimethyl formamide	anti-OS	improve EBI and cognitive dysfunction via the Keap1-Nrf2-ARE system
	telmisartan	anti-OS; inhibit cerebral vasospasm	decrease TXNIP expression
	nebivolol		increase GSH-Px activity
	curcumin		reduce TNF-α
	curcumin nanoparticles	anti-OS; anti-inflammation	keep BBB integrity; activate glutamate transporter-1; inhibit inflammation and OS
	UA		suppress the TLR4-mediated inflammatory pathway
	pterostilbene		inhibit NLRP3 inflammasome and Nox2-related OS
	apigenin	anti-OS; anti-apoptosis	inhibit EBI through the dual effects of anti-oxidation and anti-apoptosis
	peroxiredoxin1/2		
	docosahexaenoic acid		
	sodium hydrosulfide		
	cysteamine		
	gastrodin		
	naringin		
	progesterone		
	AVE 0991		decreases OS and neuronal apoptosis through Mas/PKA/p-CREB/UCP-2 pathway
	allicin		extenuate brain edema and BBB dysfunction;
	mangiferin	anti-OS; anti-inflammation; anti-apoptosis	regulate the mitochondrial apoptosis pathway, NLRP3 and NF-κB.
	memantine		inhibit inflammation-mediated BBB breakdown and ER stress-based apoptosis
	Salvianolic acid B		activate Nrf2 signaling pathway
	Salvianolic acid A		associate with Nrf2 signaling, the phosphorylation of ERK and P38 MAPK signaling
	mitoquinone	promote autophagy	activate mitophagy via Keap1/Nrf2/PHB2 (prohibitin 2) pathway
	melatonin	promote autophagy	stimulate autophagy to inhibit apoptotic death of neural cells

Moreover, the anti-OS and anti-inflammatory effects are beneficial for post-ischemic treatment. Schizandrin A inhibits inflammation and OS through the AMPK/Nrf2 pathway [172]. Tryptanthrin [173], 3, 14, 19-triacetyl andrographolide [174], quercetin [175], diosgenin [176] and irisin [177] reduce the production of proinflammatory cytokines by inhibiting the NF-κB-related signaling pathways and suppressing ROS generation. In addition, the Zinc ion chelating agents, TPEN [178], N-acetyl lysyltyrosylcysteine amide [179], Tanshinone IIA [180], resveratrol [181] and EPO-cyclosporine combination therapy [182] preserve neuronal function through anti-OS and anti-inflammatory mechanisms of action. Furthermore, many antioxidants have effects on neuroprotection via anti-OS and anti-apoptosis. Rhein [183], deuterohemin His peptide-6 [184], acteoside [185], *Radix scrophulariae* [186], pomalidomide [187], and *Clostridium butyricum* [188] protect the brain against cerebral I/R injury by inhibiting apoptosis and OS. Similarly, adiponectin [189], YiQiFuMai [190], epigallocatechin-3-gallate (EGCG) [191], *Withania somnifera* [192], SMXZF [193], and diallyl trisulfide [194] lessen neuronal impairment by attenuating mitochondrial caspase-independent apoptosis. Besides, plumbagin [187], hollow prussian blue nanozymes [195], geniposide [196], curcumin [197], and hexahydrocurcumin [198] play a role in neuronal protection by anti-OS, anti-inflammatory, and anti-apoptotic activities. In addition, as autophagy is a double-edged sword, promoting or inhibiting autophagy to exert neuroprotective effects depends on different conditions. On one hand, Sirt3 [199] and β-arrestin-1 [200] promote autophagy to play a neuroprotective role. On the other hand, vitexin [201], silibinin [202]. 3-methyladenine [203], and astragalosides [204] inhibit autophagy to protect the brain. It is also worth mentioning that isoquercetin [205, 206] and EGCG [207] alleviate brain impairment via anti-OS, anti-inflammatory, anti-apoptotic, and anti-autophagocytic properties.

Like ischemic stroke, antioxidant defenses involving OS and OS-related processes are also found in ICH and SAH. Green tea [208, 209], cofilin [210], and mammalian sterile 20-like kinase-1 [211] play a major role in ICH-induced SBI. Melatonin [51] and dexmedetomidine [212] reduce mitochondrial impairments and ameliorate SBI. Analogously, oleuropein [213] and adiponectin [214] attenuate brain edema and preserve the BBB structure in a dose-dependent manner. In addition, isoliquiritigenin [215] and Sirt3 [216] improve ICH by inhibiting inflammatory and OS activities, whereas Sodium Benzoate [217] and carnosine [218] attenuate SBI and brain edema through suppression of neuronal apoptosis. As such, metformin [219], baicalein [220], hydrogen gas [221], protocatechuic acid [222], and hypoxia-inducible factor prolyl hydroxylase domain metalloenzymes [223] prevent neurological deficits after ICH by inhibiting apoptosis, OS, and neuroinflammation in rats.

**Table 2:** Antioxidants in Depression.

	Antioxidants	Anti-OS activity	Mechanism of anti-OS activity and others
Depression	bay 60-7550	anti-OS	downregulate gp91phox; activate the cAMP/cGMP-pVASP-CREB-BDNF signaling pathway
	p-chloro-diphenyl diselenide		modulate glutamate neurotransmission
	homocysteine		inhibit ROS by activating NMDA receptors
	vitamin D		suppress OS
	2,3,5,4'-tetrahydroxystilbene-2-O-β-D-glucopyranoside	anti-OS; anti-inflammation	reduce proinflammatory factors; restore the diminished Akt signaling pathway; faciliate astrocyte proliferation and neurogenesis
	vorinostat		modulate NF-κB p65, COX-2 and phosphorylated JNK levels
	melatonin		inhibit OS and inflammation
	naringenin		
	iptakalim		
	silymarin		
	resveratrol		
	honokiol		
	oxytocin		
	vanillin		
	trigonelline		
	quercetin		
	α-tocopherol		
	baicalin		
	selenium-containing compounds		
	ketamine		increase glutamate release; affect energy metabolism
	mitochondrial uncoupling protein 2	anti-OS; anti-inflammation; anti-apoptosis	downregulate the activation of NLRP3 inflammasome; suppress the ROS-TXNIP-NLRP3 pathway in astrocytes
	dl-3-n-butylphthalide		inhibit OS, inflammatory responses and apoptosis
	indole-3-carbinol		
	25-methoxyhispidol A		
	allicin		reduce neuroinflammation, OS, iron overaccumulation; inhibit neuronal apoptosis in the hippocampus
	AVLE		suppress the apoptosis of hippocampus cells via regulation of Bcl-2/Bax pathways

Many preclinical experiments in SAH have demonstrated that inhibiting OS and OS-mediated pathological processes alleviates alleviates EBI, SBI, and neuronal deficits. Dimethyl formamide improves cognitive dysfunction via the Keap1-Nrf2-ARE system [224]. Telmisartan [225], nebivolol [226] and curcumin [227] can ameliorate cerebral vasospasm, and Pterostilbene can attenuate EBI by inhibiting the NLRP3 inflammasome [228]. Some antioxidants (e.g., apigenin [229], peroxiredoxin1/2 [230], docosahexaenoic acid [231], sodium hydrosulfide [232], cysteamine [233], gastrodin [234], naringin [60], and progesterone [235]) play a neuroprotective role in EBI through the effects of anti-apoptosis after SAH, but other antioxidants (e.g. AVE 0991 and mangiferin [56, 236] memantine [59, 237], salvianolic acid B [238, 239], salvianolic acid A [240], and allicin [241]) protect against SAH-induced oxidative injury via inhibition of oxidative, inflammatory, and apoptotic pathways. Stimulating autophagy to inhibit apoptosis of neural cells also works in improving neurological outcome with administration of mitoquinone [242] and melatonin [243].

**Table 3:** Co-antioxidants in stroke and depression from experiments.

Co-antioxidants in stroke and depression from experiments				
Antioxidants	Stroke			Depression
	Ischemic stroke	ICH	SAH	
adiponectin	✔	✔		
ECGG	✔	✔		
metformin	✔	✔		
protocatechualdehyde	✔	✔		
Sirt3	✔	✔		
curcumin	✔		✔	
DHC/A	✔		✔	
mangiferin	✔		✔	
progesterone	✔		✔	
UA	✔		✔	
dl-3-n-Butylphthalide	✔			✔
quercetin	✔			✔
resveratrol	✔			✔
baicalein		✔		✔
allicin			✔	✔
melatonin	✔	✔	✔	✔

	Clinical trials and outcomes in stroke		Clinical trials and outcomes in depression	
	Types	Outcomes	Types	Outcomes
flavonoid	meta-analysis	high flavonoid reduces risk of stroke	RCT	higher flavonoid links to lower depression risk especially among women
UA	RCT and URICOICTUS	UA is safe; UA enhances outcomes of stroke	cohort studies and meta-analysis	UA are associated with low risk of depression hospitalization and lower MDA levels
melatonin	RCT	early melatonin usage ameliorates the brain injury of asphyxial newborns	RCT	buspirone-melatonin therapy benefits cognitive function
			Review	melatonin does not affect mood disorders

### 6.2 Antioxidants in depression

Apart from stroke, using antioxidants may help improve depression. Generally, antioxidants exert antidepressant effects through their anti-OS characteristics (Table 2). Bay 60-7550 [244], p-chloro-diphenyl diselenide [245], homocysteine [246], and vitamin D [247] can attenuate depressive-like behaviors and improve depression. In a sense, the latest studies have focused on antioxidants that act against OS-induced inflammation. Examples of this include 2,3,5,4'-tetrahydroxystilbene-2-O-β-D- gluco-pyranoside [248] and vorinostat [249], which ameliorate inflammatory damage and OS to exert the antidepressant effect. Moreover, naringenin [250, 251], iptakalim [252], silymarin [253], resveratrol [254], honokiol [255], ketamine [256], melatonin [257], oxytocin [258], vanillin [259], trigonelline [260], quercetin [261], α-tocopherol [262], baicalin [263] and selenium-containing compounds [264] have neuroprotective effects in the hippocampus of mice due to their antioxidant and anti-inflammatory properties. Particularly, ketamine is involved in inducing the rapid antidepressant effects through increasing the release of glutamate in the body [265] and affecting energy metabolism in MDD [256]. Furthermore, some antioxidants with multifunctional properties demonstrate anti-OS, anti-inflammatory, and anti-apoptotic characteristics in depression. These antioxidants include mitochondrial uncoupling protein 2 [266], dl-3-n-butylphthalide [267], indole-3-carbinol [268], or 25-methoxyhispidol A [269] and allicin [270]. Of note, AVLE treatment has similar effects compared to fluoxetine on depression in the rat hippocampus [271].

### 6.3 Co-antioxidants and promising drugs in stroke and depression

Thus far, the use of antioxidants in animals has been successful in treating stroke or depression. According to the previous summarization, we have found some co-antioxidants (Table 3). Additionally, several clinical trials show that co-antioxidants exert protections in stroke and depression, including flavonoid and melatonin.

#### 6.3.1 Flavonoids

In stroke, high dietary flavonoid intake may modestly lower the risk of stroke due to its role in constraining OS-induced mitochondrial lipid peroxidation [272]. In depression, flavonoids can limit ROS production and promote the chelation of transition metal elements [273, 274]. Also, flavonoids act as reversible and competitive human monoamine oxidase inhibitors in the CNS, leading to increased central neurotransmission [275]. Moreover, a study enrolling 10,752 depressed individuals shows that higher flavonoid intake may be linked to lower depression risk, especially among older women [276].

#### 6.3.2 Uric Acid

UA is the primary endogenous antioxidant in blood. A randomized, double-blind phase 2b/3 trial (URICOI-CTUS) indicates that UA is safe without any concerns regarding safety, although the addition of UA to thrombolytic therapy does not raise the proportion of patients with excellent outcome after stroke [277]. However, a reanalysis of the URICOICTUS trial suggests that UA suppresses infarct growth, and is more effective than the placebo in reaching an excellent outcome in patients who are treated with alteplase following acute ischemic stroke [278]. Another clinical study supports the efficacy of UA therapy by showing decreased infarct growth and enhanced outcome in stroke survivors [279]. In depression, researchers have found that UA is associated with a low risk of hospitalization for depression after examining the plasma levels of UA in 96,989 depressed subjects [280]. Moreover, the efficacy of UA in depressed patients is supported by another large-scale study [281].

#### 6.3.3 Melatonin

To our knowledge, few clinical trials have studied the neuroprotective effects of melatonin on cerebral ischemia prevention. A prospective trial involving 45 neonates indicates that it is practicable to provide early administration of melatonin to ameliorate brain injury in a choking newborn infant [282]. In regards to treatment of MDD, preliminary findings of a study reveal that a combination of buspirone with melatonin can partially benefit cognitive function [283].

**Table 4:** Antidepressants in PSD treatment.

Antioxidants in PSD	Clinical trials	Outcomes
fluoxetine	FOCUS	not support routine use of fluoxetine in preventing PSD or promoting function recovery
fluoxetine/paroxetine	meta-analysis of 12 trials	fluoxetine is the worst choice for PSD treatment; paroxetine is the best drug in terms of efficacy and acceptability
	meta-analysis of 20 RCTs	citalopram has similar efficacy and safety as other SSRIs but acts faster than them
fluoxetine	FLAME	exhibit a positive connection between motor recovery
escitalopram	Cochrane review	escitalopram is the best tolerated SSRI, followed by sertraline and paroxetine for PSD
escitalopram	RCT	not take effects on depressive symptoms; diarrhea is more likely to occur
escitalopram	RCT	effective at decreasing the incidence of depression in nondepressed patients
Citalopram	RCT	safe for patients with acute ischemic stroke
Citalopram	RCT	different effects in different stages of PSD
citalopram	RCT	SSRI treatment is well tolerated and beneficial in PSD
SSRI	registry-based score-matched follow-up study	pre-stroke SSRI use increases risk of the hemorrhagic stroke; no increased stroke severity and mortality ischemic stroke
milnacipran	RCT	milnacipran prevents post-stroke depression; safe to use without serious adverse events

Although many co-antioxidants are found in animal models of both diseases, only a few clinical trials have confirmed the efficacy of antioxidants in stroke and depression. The difficulty of antioxidants acting through the BBB in patients may be the reason for this result. Nevertheless, OS and OS-mediated pathways are co-mechanisms of stroke and depression. So, what is the role of antidepressants in stroke survivors?

### 6.3 Antidepressants in post-stroke

At present, antidepressant drugs are widely used for treating post-stroke depression (PSD), but there are many adverse reactions at the same time. These events include gastroenterological symptoms, falls/fracture, and epilepsy [284]. Therefore, future clinical trials should emphasize their focus on the efficacy and safety of antidepressant drugs in preventing or treating stroke (Table 4). Herein, we summarize some clinical trials in recent years with the objective of determining whether antidepressants (e.g., fluoxetine, escitalopram, milnacipran) are necessary for PSD patients. Results of FOCUS (effects of fluoxetine on functional outcomes after acute stroke) represent a lower incidence of new depression and a higher risk of bone fractures in the group in which fluoxetine is allocated, when compared with the controls. Those outcomes do not support the routine use of fluoxetine in preventing PSD or promoting function recovery [285]. In addition, a meta-analysis of 12 suitable trials demonstrated that fluoxetine is the worst choice for PSD treatment, while paroxetine is an effective and acceptable drug [286]. However, FLAME (fluoxetine for motor recovery after acute ischemic stroke) exhibits a positive connection between motor recovery and fluoxetine use in PSD patients [287]. The second drug is escitalopram. Kim K et al. did a Cochrane systematic review of 13 trials of antidepressant drugs, and confirmed that escitalopram is the most tolerated SSRI [288]. Another study also indicates that escitalopram was well-tolerated, though it does not have any effects on the depressive symptoms in PSD patients [289]. Robinson et al. illustrated that escitalopram is significantly effective at decreasing the incidence of depression in patients with recent stroke over 12-month therapy [290]. Thirdly, citalopram is reported to be a safe medication in patients with acute ischemic stroke [291], and it improves PSD [292]. In conclusion, fluoxetine is not necessary for PSD treatment, but it is good for motor recovery. Escitalopram and citalopram are both tolerated and may have benefits in PSD patients. However, the question of whether to allocate antidepressants in post-stroke patients is still controversial, when considering efficacy and safety. Additionally, is pre-stroke SSRI therapy useful for patients with stroke? Mortensen et al. conducted a study enrolling hemorrhagic stroke patients and ischemic stroke patients. They found that pre-stroke SSRI use increased severity and mortality in patients with hemorrhagic stroke, but there was no increase in ischemic stroke patients [293]. Finally, milnacipran also plays a role in preventing PSD and it is safe to use, without any serious adverse events, according to an RCT performed by Ching-Shu Tsai [294]. Due to the limited data showing a relationship between depressants and PSD, more multicenter clinical trials should be conducted.

## DISCUSSION

This review discusses the central role of OS in the “aging-stroke-depression” network and antioxidants in treating stroke or depression, as well as antidepressant in PSD. Although all of the studies have immeasurable contributions to scientific exploration, there are some limitations in the current studies. First, depression is a psychiatric disorder lacking effective biomarkers or methods, and depressive symptoms may be less apparent, making it difficult to procure an accurate diagnosis for depression [10]. Relatively uniform and accurate diagnostic criteria for depression must be improved [295]. Second, this review focuses on major depression, leaving an unclear relationship between other depression phases and stroke. Third, although this review suggests aging as a risk for stroke and depression, the link is often found in aged adults, but not analyzed in other age groups [296, ]33]. For future studies, it is suggested that there should be an emphasis on certain age demographics. Fourth, human brains have a natural and complex protective barrier, which prevents antioxidant drugs from entering into the CNS, partially explaining the reduced efficacy of these drugs in experimental models [297]. Finding carriers that can penetrate the blood-brain barrier, such as nanoparticles, may address this problem. Fifth, there are data deviations due to the lack of dose-response analysis, limitations on sample size, representativeness of the sample, statistical methods, inclusion criteria, and follow-up [37, 298, 299]. Therefore, studies should be conducted in a more precise manner. Furthermore, drug dose, window time, and methods of administration also warrant our attention. All things considered, further studies are required to improve the diagnosis of depression, to find drugs directed at overproduced ROS with higher efficacy and safety, and to enhance the quality of life after diseases.
